# Supplementary material for: Epigenetic Clock Explains White Matter Hyperintensity Burden Irrespective of Chronological Age
Source: Biology (Basel). 2022 Dec 24;12(1):33. doi: 10.3390/biology12010033 (PMC9855342; doi:10.3390/biology12010033)
Supplement: Supplementary file 1 [file biology-12-00033-s001.zip › biology-2074370-supplementary.pdf]

Supplementary Table S1: Effects of main covariables on Log-WMH

Variable		r/mean(SD)	p-value
**Age**		0.392	<0.001
**Sex**			
	Female	2.08 (1.07)	0.247
	Male	2.24 (1.09)	
**Smoking habit**			
	No	2.29 (1.03)	0.03
	Yes	1.97 (1.16)	
**Alcohol consumption**			
	No	2.29 (1.05)	0.01
	Yes	1.89 (1.13)	
**Hypertension**			
	No	1.78 (1.19)	0.002
	Yes	2.3 (1.03)	
**Diabetes**			
	No	2.03 (1.11)	0.011
	Yes	2.39 (1.01)	
**Dyslipidemia**			
	No	2.12 (1.06)	0.466
	Yes	2.22 (1.1)	
**Body mass index**		-0.084	0.203
**Atrial fibrillation**			
	No	2.2 (1.1)	0.657
	Yes	2.13 (1.04)	
**Previous myocardial infarction**			
	No	2.17 (1.09)	0.740
	Yes	2.25 (1.03)	
**TOAST**			
	Atherothrombotic	2.07 (1.03)	0.117
	Lacunar	2.34 (1.12)	
	Cardioembolic	1.99 (1.01)	
	Undetermined	2.29 (1.16)	

Each row represents the association between an explanatory variable and log WMH. Values represent Pearson correlation coefficient for continuous variables and mean (SD) for categorical variables (second column). The third column represents the significance in correlations or differences between groups, and *p*-values have been obtained with Pearson correlation coefficient, *t*- or anova-tests according to the type of each variable.
